# The relationship between Japanese adults’ age and self-reported verbal strategies when lying

**DOI:** 10.3389/fpsyg.2022.1075239

**Published:** 2023-01-16

**Authors:** Naoya Tabata, Aldert Vrij

**Affiliations:** ^1^Department of Policy Studies, Aichi Gakuin University, Nisshin, Japan; ^2^Department of Psychology, University of Portsmouth, Portsmouth, United Kingdom

**Keywords:** lies, verbal strategies, age, adults, high-context culture, Japanese

## Abstract

We examined the relationship between age and self-reported verbal deception strategies in Japanese adults. Japanese participants (*N* = 153) aged 18 to 73 years took part in this study. We requested the participants to state their age and freely describe how they structure their speech to appear convincing when lying during their daily interactions. We extracted 13 verbal strategies from the participants’ open-ended descriptions. Japan is a high-context culture. The results indicated that 11 categories corresponded to the verbal strategies reported in previous studies on lying conducted in low-context cultures. However, two strategies mentioned in the current study, making ambiguous statements and adding irrelevant details to the lie, were not reported in low-context cultures. As expected, age was significantly and negatively correlated with the number of verbal strategies used when lying. Moreover, verbal strategies that seem relatively cognitive demanding were used less as the age of the participants increased. We concluded that these results reflected the age-related decline of cognitive abilities.

## Introduction

Research suggests that verbal cues are more effective for detecting deception than nonverbal cues (e.g., DePaulo et al., 2003; Vrij, 2019; Vrij et al., 2019, 2022). Specific interview techniques, including the Strategic Use of Evidence (SUE; Hartwig et al., 2014), the Verifiability Approach (VA; Nahari, 2018), and Cognitive Credibility Assessment (CCA; Vrij et al., 2017) have been developed to elicit verbal cues to deception.

Verbal deception strategies, or how people express what they want to say when they lie, are critical elements of verbal deception. It has been argued that focusing on the verbal strategies behind individual statements contributes to a general understanding of verbal behavior when lying (DePaulo et al., 2003; Vrij et al., 2010). A better understanding of lie tellers’ verbal strategies could also be used to develop specific interview techniques aimed to counteract these strategies (Vrij and Granhag, 2012). In fact, the specific interview techniques developed to date (SUE, VA, and CCA) all aim to exploit the verbal strategies lie tellers use. Research conducted in Sweden and the United States has focused on verbal deception strategies used (Strömwall et al., 2006; Hartwig et al., 2007, 2010; Hines et al., 2010; Strömwall and Willén, 2011). The participants of these studies self-reported in response to an open-ended question the things they say or avoid saying to sound convincing during mock interrogations, which were coded to establish data-driven categories of verbal strategies.

Identifying factors influencing verbal deception strategies could result in a better understanding of these strategies. One critical factor is the communication style, defined as how people communicate with others (Hall, 1976; Liu, 2016). Cultures have been categorized according to whether people rely more on language or more on context for communication (Liu, 2016). Low-context cultures use a communication style that relies heavily on language. In contrast, high-context cultures use a communication style that relies heavily on context. According to Liu’s (2016) classification, the United Kingdom is a low-context culture, and Japan is a high-context culture. Tabata and Vrij (2022) compared self-reported verbal strategies used to appear convincing when lying and truth-telling between British and Japanese participants. Deception research has been conducted mainly in the so-called WEIRD (Western, educated, industrialized, rich, and democratic; Gerlach et al., 2019) cultural groups, and this study was an exception to this trend. Tabata and Vrij (2022) asked participants to rate how much they endorsed 16 self-reported verbal strategies that lie tellers and truth tellers use to appear convincing. They extracted these 16 strategies from previous studies all conducted in low-context cultures using an open-ended questions method (Strömwall et al., 2006; Hartwig et al., 2007, 2010; Hines et al., 2010; Strömwall and Willén, 2011). The results of this closed-questions method questionnaire revealed that British participants were more likely than Japanese participants to try to tell a lie in a logical way and to focus on facts, which corresponded to differences in communication styles between the two countries. For example, British participants were more concerned with providing innocent reasons and avoiding/denying incriminating evidence when lying than when truth-telling, which was not the case for Japanese participants. Tabata and Vrij (2022) also reported that the Japanese were less likely than the British to self-report using verbal strategies based on Grice’s cooperative principles (1975). The Grice cooperative principles describe how people achieve effective conversation in general social situations. Violating Grice’s cooperative principles (1975) is considered deceptive in low-context cultures, where language dominates when communicating with others (McCornack, 1992; McCornack et al., 1992), but those principles are often disregarded in high-context cultures (He, 2012; Herawati, 2013; Al-Qaderi, 2015). Indeed, coders in a study of Japanese participants (Tabata, 2009) categorized 9 of 55 participants (16.4%) as using the “Make the story ambiguous” strategy when they were forced to lie in experimental situations, which violated the maxim of manner (Grice, 1975).

The current study focused on age as another possible factor related to verbal deception strategies. A series of studies on the theory of mind, the ability to understand that other people have thoughts, knowledge, and feelings that are not the same as ours (e.g., Premack and Woodruff, 1978; Wellman et al., 2001), suggest that people’s ability to conduct complex deception develops with the development of the theory of mind, which enables to use complex verbal deception strategies (e.g., Talwar and Lee, 2008). However, the relationship between age and verbal strategies when lying used by adults who have acquired the theory of mind remains unclear.

Lying can be mentally taxing (e.g., Zuckerman et al., 1981; Vrij, 2008; Christ et al., 2009). It is also known that cognitive abilities decline with age (e.g., Tucker-Drob, 2011). As a result, age-related cognitive changes might affect verbal strategies when lying. Studies of neurobiological variables including regional brain volume showed that continuous age-related decline begins in the 20s (e.g., Pieperhoff et al., 2008), resulting in age related cognitive decline. Normal cognitive aging begins relatively early in adulthood in healthy adults (Salthouse, 2019), and specific cognitive abilities such as reasoning and speed start to decline as early as from 20 or 30 years of age (Salthouse, 2009). Several studies have shown the effects of age-related decline of cognitive abilities on lying-related behaviors and judgments. For example, the number of lies told was negatively associated with age (Serota et al., 2010); and older adults were worse at lying or detecting lies than younger adults, and these detection failures were mediated by the relationship between age and older adults’ decline in recognizing emotions (Ruffman et al., 2012). Based on these findings, we hypothesized that age would affect verbal deception strategies, such that fewer verbal strategies would be used as a person ages, reflecting the decline in older adults’ cognitive abilities.

Tabata and Vrij (2022) asked participants to complete a verbal deception strategy questionnaire which included verbal strategies based on studies using the open-ended question method conducted in Sweden and the United States, which are classified as low-context cultures (Liu, 2016). Tabata and Vrij (2022) could thus compare differences in Japanese and British participants in endorsing verbal strategies identified in low-context cultures but did not give Japanese participants the opportunity to report strategies that are unique to their culture. In the current study we used the open-ended question method to identify verbal strategies used by Japanese participants when lying. Unlike the closed-ended question methods used by Tabata and Vrij (2022), an open-ended question method has the advantage that respondents can report new verbal deception strategies (e.g., Vrij, 2008).

## Method

### Participants

Japanese adults (*N* = 153, 82 men and 71 women; mean age 26.01 years, *SD* = 11.51, age range 18 to 73 years; Age distribution, 20 participants in their teens, 80 in their 20s, 20 in their 30s, 10 in their 40s, 13 in their 50s, and 10 in their 60s or older) took part in this study on a voluntarily basis. The participants’ age distribution was skewed (Skewness = 1.92, Kurtosis = 2.66). Therefore, we used the log-transformed age in the analysis because the Kurtosis exceeded ±2, and the normality assumption of the distribution was not satisfied (e.g., Kunnan, 1998).

### Procedure

We conducted this survey in July and August of 2021 after a university class on social psychology and at a public lecture on library information science for citizens held at the same university. We distributed questionnaires to the participants at the same time after the class or the lecture. The participants indicated their age and gender, and then we asked the participants the following. “Please explain the expressions you use when you lie in your daily interactions. Please freely describe how you structure your speech to appear convincing in your daily interactions. Please give as many strategies as you can think of.” We gave participants 5 min as sufficient time to recall their behavior. We instructed the participants who did not use any verbal strategies to answer that they did not use a specific strategy. We debriefed the participants after collecting the questionnaires.

### Response coding

The participants’ open-ended descriptions of the verbal strategies they used to appear convincing were first analyzed qualitatively by three coders, who were two Japanese undergraduate students majoring in social psychology and the first author. All of them were native Japanese speakers. We first excluded 33 descriptions of the 337 descriptions made by the 153 participants that were unrelated to verbal strategies. Then, we categorized the remaining 304 descriptions obtained from 143 (94.5%) participants so that the responses corresponded with the verbal strategies described in Tabata and Vrij (2022). The responses that did not correspond to Tabata and Vrij were categorized as new categories in a data-driven manner. Table 1 shows the 13 verbal strategies that we categorized. Eleven categories were identical to Tabata and Vrij, whereas two categories, “Make the story ambiguous” and “Add irrelevant details,” were new categories. We defined “Make the story ambiguous” as blurring the content of a statement, and “Add irrelevant details” as adding details unrelated to the lie. “Make the story ambiguous” violated the maxim of manner, and “Add irrelevant details” violated the maxim of relevance (people should keep to the point) in Grice’s cooperative principles (1975).

**Table 1:** Verbal strategy categories used when lying and definitions.

Category	Definition
Deny/Avoid incriminating details	Avoiding reporting incriminating details while giving more details about innocent elements of the story.
Obey the maxim of manner	Avoiding obscurity and ambiguity and being brief and orderly.
Minimal detail	Saying as little as possible so that if the story needed to be repeated there would be less room for error.
Rich in detail	Giving as much detail as possible about what has happened.
Plausibility	Giving a statement that sounds plausible (that sounds as if it really could have happened).
Coherent and consistent	Explaining everything the same way even if asked the same question again.
Make the story ambiguous	Blurring the content of a statement.
Add irrelevant details	Adding details unrelated to the lie.
Provide innocent reason	Providing an innocent reason for an activity.
No hesitation	Appearing decisive.
Unrehearsed story	Making the story sound spontaneous.
Consistent story	Sticking with a story and do not change elements within it.
Emotions	Explaining the feelings experiencing during the event.

Next, the two Japanese undergraduate students allocated each description to the 13 categories. The agreement rate between the two classifications was 91.8% (*κ* = 0.91). The discrepancies were resolved in a discussion between the two coders.

## Results

We included the 10 (6.5%) participants who indicated they did not use a specific verbal strategy in the analyses. Table 2 shows the number of times each verbal deception strategy was mentioned and the percentage of participants who reported them. We can see that “Deny/Avoid incriminating details strategy” was most frequently mentioned (56 times), followed by “Obey the maxim of manner” (44 times), “Minimal detail” (43 times), and Rich in detail (41 times).

**Table 2:** Verbal strategies when lying, the number of statements, percentage of participants using statements, and correlation with log-transformed age (*N* = 153).

Verbal strategies	Number of statements	Participants who stated the statement (%)	Correlation with log-transformed age		
when lying			*r*	*p*	95% CI
Deny/Avoid incriminating details	56	32.0	−0.27	<0.001	[−0.41, −0.12]
Obey the maxim of manner	44	25.5	−0.19	0.022	[−0.33, −0.03]
Minimal detail	43	23.5	−0.02	0.852	[−0.17, 0.14]
Rich in detail	41	25.5	−0.25	0.002	[−0.39, −0.09]
Plausibility	24	15.0	−0.10	0.243	[−0.25, 0.06]
Coherent and consistent	23	15.0	−0.09	0.256	[−0.25, 0.07]
Make the story ambiguous	19	12.4	−0.05	0.510	[−0.21, 0.11]
Add irrelevant details	16	10.5	−0.17	0.036	[−0.32, −0.01]
Provide innocent reason	14	9.2	0.14	0.084	[−0.02, 0.29]
No hesitation	13	6.5	0.01	0.942	[−0.15, 0.16]
Unrehearsed story	7	4.6	−0.02	0.801	[−0.18, 0.14]
Consistent story	3	2.0	0.07	0.399	[−0.09, 0.22]
Emotions	1	0.7	−0.00	0.974	[−0.16, 0.16]

We scored whether a participant mentioned using each verbal strategy category (Mentioned = 1 and not Mentioned = 0). On average participants reported 1.82 (*SD* = 1.03) categories, which was significantly and negatively correlated with the log-transformed age of the participants (*r* = −0.40, *p* < 0.001, 95% CI [−0.53, −0.26]). Table 2 shows the correlation results for each category. Four categories – “Deny/Avoid incriminating details,” “Obey the maxim of manner,” “Rich in detail” and “Add irrelevant details” – were significantly and negatively correlated with age.

## Discussion

This study investigated the relationship between self-reported verbal deception strategies and age in Japanese adults using the open-ended question method. The results supported our hypothesis based on the decline of cognitive abilities with age. Age was significantly and negatively correlated with the number of verbal deception strategies used.

Moreover, the verbal deception strategies significantly and negatively related to age – “Deny/Avoid incriminating details,” “Obey the maxim of manner,” “Rich in detail” and “Adding irrelevant details” – can all be considered to be relatively cognitively demanding to execute. Denying or avoiding incriminating details requires fabricating a denial that conforms to known facts (Hartwig et al., 2010), which is mentally taxing. Obeying the maxim of manner requires stories with complex speech structures to avoid ambiguity (Grice, 1975). Telling a story rich in detail or adding irrelevant details requires to make up details and fabricating details can be cognitive demanding (e.g., Köhnken, 2004; Vrij, 2008). These results corroborate the idea that older participants may find it challenging to use these verbal strategies. However, since we did not measure cognitive abilities, this remains an empirical question that needs to be examined. This study revealed two new verbal strategies – “Make the story ambiguous” and “Add irrelevant details” – that have not been identified in low-context cultures using the open-ended question method. Participants in low-context cultures most likely avoid these strategies because they violate Grice’s cooperative principle and violating this principle sounds suspicious (McCornack, 1992; McCornack et al., 1992). However, speakers in high-context cultures often disregard Grice’s cooperative principle (He, 2012; Herawati, 2013; Al-Qaderi, 2015). Over 10% of the participants in this study mentioned using these two verbal deception strategies, suggesting that they are common in Japan. The use of the open-ended question method in high-context participants contributed to discovering these new verbal deception strategies.

Another finding of this study was that “Deny/Avoid incriminating details” were most common strategies when lying in Japanese adults, followed by “Obey the maxim of manner,” “Minimal detail” and “Rich in detail.” “Minimal detail” and “Rich in detail” seems to contradict each other. Which of these two strategies people favor may depend on the context or the personality of the lie teller. The results of this study imply that age is a significant factor in the verbal deception strategies. Tabata and Vrij (2022) pointed out the lack of research on verbal deception strategies and the present study helps clarifying the verbal deception strategies used by younger and older adults. If our finding, older adults use simpler strategies than younger adults, will be replicated in future research a next step could be to develop specific interview protocols for younger and older adults aimed to counteract the specific strategies they use.

Several limitations of this study should be noted. First, the study examined the relationship between self-reported verbal deception strategies and age only in Japanese participants. Because we examined a topic never examined in verbal deception strategies research before (the relationship between self-reported verbal deception strategies and age), we started small scale with only one high-contact culture country. Since the predicted relationship between verbal deception strategies and age emerged in that high-context culture country (Japan), future research could examine whether this will be replicated in other high-context cultures. If so, it will make the findings more robust. Conducting research into examining the relationship between verbal deception strategies and age in low context countries is also essential. Based on Tabata and Vrij (2022), we can assume that people in low-context cultures are more accustomed to lying logically than people in high-context cultures, so lying may be less mentally taxing in low-context cultures than in high-context cultures. A negative relationship between age and self-reported verbal deception strategies may thus be most pronounced in high-context cultures. Second, in the current study we focused on lying in general social situations. For a more comprehensive understanding of verbal deception strategies, it is desirable to include more specific contexts, since different verbal deception strategies may be used in different contexts. Third, we only studied participants up to their early 70s in age but cognitive decline may be more pronounced in adults older than 70 years. A study that includes participants older than those in the current study might shed more light on age-related patterns of cognitive decline when using verbal deception strategies. Fourth, we measured self-reported verbal strategies rather than verbal responses. Focusing on verbal strategies used for lying facilitates insight into the lie teller’s thought processes (Vrij et al., 2010). However, it is unclear how the actual speech content reflects the verbal strategy. We suggest future studies to empirically examine the link between self-reported verbal deception strategies and verbal deceptive behavior. Fifth, the open-ended question method has as limitation that it only reveals strategies participants could think of and reported. Participants may also use verbal deception strategies that they did not mention (e.g., Vrij, 2008). It would thus be desirable to examine the correlation between verbal deception strategies and age using the closed-ended question method, including the new strategies obtained in this study. Finally, despite the evidence from several studies that age-related decline of cognitive abilities affect lying-related decisions and behaviors (e.g., Serota et al., 2010; Ruffman et al., 2012), the findings of this study might be explained by mechanisms other than cognitive decline. For example, older participants might have stopped using verbal deception strategies they have found to be less effective. We have not addressed which verbal strategies are effective for successful deception because we assumed it was dependent on the situation (e.g., Levine, 2022). Further studies could investigate the possibility that specific verbal strategies, especially those that complicate the structure of a story, might be intentionally unused due to reasons other than age.

In summary, this study demonstrated that age was significantly and negatively correlated with the number of verbal deception strategies. Particularly, verbal strategies that complicate the structure of a story tended to be less used as participants’ age increased. We hope that this study encourages other researchers to examine the relationship between age and self-reported verbal deception strategies.

## Data availability statement

The raw data supporting the conclusions of this article will be made available by the authors, without undue reservation.

## Ethics statement

The studies involving human participants were reviewed and approved by Ethical Committee for Policy Studies Association in Aichi Gakuin University. The patients/participants provided their written informed consent to participate in this study.

## Author contributions

NT performed the data collection and analysis and wrote the first draft of the manuscript. AV contributed to the manuscript’s final draft and revisions and read and approved the final version of the manuscript for submission. All authors contributed to the article and approved the submitted version.

## Funding

This study was funded by a research grant from the Institute for Policy Science at Aichi Gakuin University.

## Conflict of interest

The authors declare that the research was conducted in the absence of any commercial or financial relationships that could be construed as a potential conflict of interest.

## Publisher’s note

All claims expressed in this article are solely those of the authors and do not necessarily represent those of their affiliated organizations, or those of the publisher, the editors and the reviewers. Any product that may be evaluated in this article, or claim that may be made by its manufacturer, is not guaranteed or endorsed by the publisher.
